# Supplementary material for: Bone Modeling after Orthodontic Extrusion: A Histomorphometric Pilot Study
Source: J Clin Med. 2022 Dec 9;11(24):7329. doi: 10.3390/jcm11247329 (PMC9781502; doi:10.3390/jcm11247329)
Supplement: Supplementary file 1 [file jcm-11-07329-s001.zip › jcm-1959252-supplementary.pdf]

Table S1. Teeth-related features.

TOOTH n=12	Bone tissue (%)	Connective tissue (%)	Mineralized bone (%)	Osteoid tissue (%)	Woven bone (%)	Lamellar bone (%)	Trabecular bone (%)	Bone marrow (%)
**38**	100	0	80	20	0	80	0	20
**48**	100	0	70	30	40	30	0	0
**38**	90	10	80	10	70	10	0	0
**48**	100	0	100	0	90	10	0	0
**48**	100	0	90	10	20	80	0	0
**38**	80	20	70	10	20	70	0	0
**48**	100	0	100	0	0	100	0	0
**48**	100	0	60	40	10	0	50	0
**48**	80	20	90	5	30	69	0	0
**48**	100	0	90	10	10	80	0	0
**38**	100	0	50	50	10	40	0	0
**38**	100	0	80	20	0	80	0	0
